# Worker Allometry in Relation to Colony Size and Social form in the Fire Ant *Solenopsis invicta*

**DOI:** 10.1673/031.010.9401

**Published:** 2010-07-06

**Authors:** Matheus B. Araujo, Walter R. Tschinkel

**Affiliations:** Department of Biological Science, Florida State University, Tallahassee, FL 32306-4370

**Keywords:** imaginai discs, isometry, growth rate, nutrition, polymorphism, size and shape

## Abstract

Workers of the polymorphic fire ant *Solenopsis invicta* Buren (Hymenoptera: Formicidae) show modest changes of shape with increases in body size. These shape changes (allometries) have been described only for workers taken from mature colonies of the monogyne social form. For the study reported here, workers were collected from small and large monogyne and large polygyne colonies for tests of the effects of colony size and social form on allometry. The differential growth of body parts in relation to total body growth was determined by measurement of all major body parts and regression of the logs of these measurements, or their ratios, on the log of the body size. The slopes of these regressions defined the allometric relationships, and the slopes for these three types of colonies were compared for determination of the influence of colony size and social form on allometric rules. Most allometric constants did not differ with colony size or social form, but head shape, relative antennal size, and alinotum shape did. For a given worker size, heads of workers from small monogyne colonies or from polygyne colonies were narrower above the eyes. Antennae of workers from large monogyne colonies were relatively shorter than those from small monogyne or polygyne colonies (which did not differ). Alinotum heights of small workers from small monogyne colonies were greater than those from large monogyne or polygyne colonies (which were isometric and did not differ). These observed differences in allometric constants suggest that the relative growth rules are not completely determined by worker body size but are affected by colony size and social form. These differences are discussed in light of the growth of imaginai discs under conditions of fixed resources.

## Introduction

Worker ants that are represented by a wide range of body size and shape within a single colony are said to be polymorphic (Wilson 1953). Only 15–20% of ant genera include polymorphic species (Hölldobler and Wilson 1990; Powell and Franks 2006). In polymorphic ants, this variation of worker size and shape has been shown to be associated with division of labor, presumably because particular sizes and morphologies are better suited for particular tasks (Wilson 1971; Hölldobler and Wilson 1990; Tschinkel 2006; Schöning et al. 2005). The fire ant *Solenopsis invicta* Buren (Hymenoptera: Formicidae) is a widespread polymorphic species adapted for inhabiting disturbed areas (Tschinkel 2006).

In polymorphic species, different relative growth rates of body parts in relation to each other, or in relation to the whole body, result in size-related differences of shape readily described through morphometry. Most studies have used morphometry to ask specific questions related to functional hypotheses and have therefore used only a few body measurements, primarily head measurements. In contrast, Tschinkel et al. (2003) searched more generally for growth-related shape changes and therefore measured all of the most biological meaningful allometric relationships. They found that heads changed from barrel shaped in small workers to heart shaped in large ones. Similarly, antennae became relatively shorter as workers became larger, the alinotum more robust, and the gaster relatively larger. Several measures, including head length and leg length, remained in constant proportion to body length in all sizes of workers.

The relationship between the body sizes of workers and the shapes of their parts has been well described for a number of ant species many times (Hölldobler and Wilson 1990; Tschinkel 2006), but whether or not these relationships can also be affected by factors outside the worker's body has not often been considered. A recent exception is the study by Kenne et al. (2000), who used the slope of the scape vs. the head width of *Myrmicaria opaciventris* to show that the allometry changed from negative in workers from incipient colonies founded by single queens to isometric in those from colonies founded by multiple queens and to positive in larger colonies. In this species worker allometry depends therefore not only on the size of the worker itself but also on queen number and colony size. Tschinkel et al. (2003) collected workers only from mature, monogyne colonies, so the relationship among allometry, colony size, and social form in this species remains unknown. Do individual worker growth rules change with colony size and/or social form, or is worker shape a function only of worker size and not of colony size or social form? Comparing the allometry of small monogyne colonies to that of large ones permits determination of whether colony size affects worker size and shape. By the same token, the comparison of large monogyne colonies with similar-sized polygyne colonies will reveal whether social form has any effect on worker growth rules. The study reported here potentially yields insights into the development and evolution of worker polymorphism.

## Materials and Methods

We determined the size/shape relationship of workers from small and large monogyne and from large polygyne colonies by linear regressions of a set of body measurements. Workers from three small monogyne, three large monogyne, and three large polygyne colonies of *S. invicta* were collected into 70% ethanol in Tallahassee, Florida. Colony size was estimated from the mound volume (Tschinkel 1993; Tschinkel et al 1995). Small monogyne colonies all had mound volumes less than 0.14 L, whereas those of large monogyne and polygyne colonies all exceeded 10 L. Before workers were collected from polygyne colonies, the presence of multiple queens attractive to workers (i.e., reproductively active) was first confirmed.

From ethanol, workers were transferred into well plates, one worker per well, and were dried in a 40° C oven for 2 days. Workers were chosen somewhat nonrandomly; very small and very large workers were overrepresented so as to increase the power of the regression analysis. The dry weight was measured on a Cahn microbalance with a precision of 1.0 µg. After weighing, the dry ants were humidified in a refrigerator for 2 days so that they would be less brittle during dissection. They were then carefully dissected and the parts of each worker placed within a small rectangle on a card covered with double-stick tape. Each rectangle corresponded to the visual field of a Nikon Microphot-FX equipped with Olympus DP-10 digital camera. At the same time and magnification, a 2-mm scale was photographed. From these images, 25 worker parts were digitally measured by means of ImageJ software, calibrated with the image of the 2-mm scale.

### Data analysis

The 25 measurements of each ant were compiled, log-transformed, and subjected to ordinary least squares (OLS) linear regressions with STATISTICA 6.0. At the urging of a reviewer, reduced major axis regression (RMA) was performed on the log-transformed data with a program by A. J. Bohonak and K. van der Linde (http://www.kimvdlinde.com/professional/rma.html). Because application of RMA did not change any conclusions about differences among colony types, OLS is the basis of this report. A comparison of the parameters for OLS and RMA is presented in Appendix 4 (appendices available online).

Colonies within colony type were tested for differences of regression parameters, and although these were occasionally present, they were smaller than the differences between types. Data from the three colonies within each type were therefore pooled for the regressions comparing colony types. Outliers more than two standard deviations from the mean were excluded after residual analysis.

We estimated total body length by summing HL, AL, PL1, PL2 and GL, as did Tschinkel et al. (2003). To avoid ambiguous measurements due to the telescoping nature of the gaster, only its first segment was included in the body-length measurement. Our “body length” is therefore not equivalent to the true body length (Tschinkel et al. 2003).

The log-transformed body measurements and their ratios to body length were regressed on total body length. The slope of these regressions estimated the relative growth rates; a slope of 1.0 indicated isometry (equal growth rates). Slopes greater than 1.0 indicated positive allometry, and slopes less than 1.0 negative allometry. For easier comparison, we regressed the ratio of body parts on total body length. When the shape (i.e., ratio) changes with body size, such regressions of ratios have slopes significantly different from zero. The slopes of significant regressions of different colony types were compared by means of the standard error of the slope estimate and *t*-tests; an alpha level of 0.05 was used to reject the null hypothesis of no difference. Those slopes in which at least two colony types differed are the basis of this paper.

### Preliminary procedures

Allometric constants for large monogyne colonies were first tested against those of Tschinkel et al. (2003) to determine whether our results were comparable.

Next, nonisometric relationships were tested for differences of slopes between the colony types, that is, whether, even if a body part grew at a rate different from that of the whole body in all three colony types, types did so at significantly different rates.

## Results

### Preliminary procedures

The differences between our allometric constants for large monogyne colonies and those of Tschinkel et al. (2003) were few and small, indicating that methods and scales were similar and that the two studies detected the same allometries.

**Figure 1.**
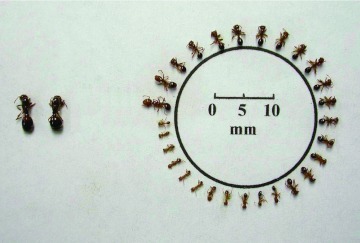
Polymorphism of polygyne *Solenopsis invicta*; notice multiple queens to the left. This presentation is in homage to that of Porter and Tschinkel (1985). High quality figures are available online.

The following sections describe only major body parts that were allometric *and* in which at least two of the three colony types differed significantly in growth rates (slopes). The parameters for all regressions within colony types are shown in Appendices 1–3. The polymorphism in polygyne colonies is shown in Figure 1.

### Differences in the size ranges of workers

**Figure 2.**
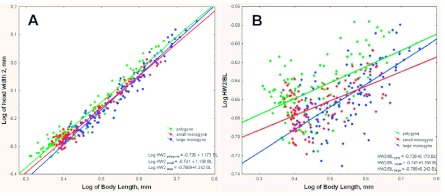
(A) Although both polygyne and monogyne colonies displayed positive allometry in head width above the eyes, workers from large monogyne ants had a higher growth rate (1.23, as opposed to 1.16 for small monogyne and 1. 17 for polygyne) in relation to the total body size. Moreover, note that polygyne colonies have more very small workers and fewer very large workers; i.e., the entire distribution is shifted toward smaller size. (B) Plotted as a log HW2-to-BL ratio against BL, the differences in positive allometry become clearer. High quality figures are available online.

Figure 2A shows that the smallest workers of polygyne colonies were about 2 mm in body length, smaller than those of the small monogyne colonies (2.3 mm), which in turn were smaller than those of the large monogyne ones (2.5 mm). The differences are not as clear in the upper range of worker size, but large monogyne colonies had the largest workers (up to 5.5 mm body length). Small monogyne colonies had few workers with body length greater than 4.5 mm, and polygyne colonies were intermediate between the two.

### Differences in size-related shape change

Workers of the same body length did not have the same shapes in the three colony types, differing primarily in the shape of the head, the antennal length, and the shape of the alinotum. The ratio of head width above the eyes (HW2) to body length (BL) was used to estimate changes in head shape [head length (HL) was not considered further here because it is isometric to body length and therefore mathematically redundant with it (note that Table 1, lines 2 and 3 are similar; Table 2, lines 3 and 4 are similar]. Workers of a given size that had higher HW2/BL ratios had heads that were less barrel-shaped and more heart-shaped, that is, relatively wider above the eyes. The slopes of the regressions of HW2 on BL and HW2/BL on BL (log-log) indicated how rapidly this shape change occurred as workers became larger. Figures 2A, B show that, compared to those of workers from polygyne and small monogyne colonies, the heads of workers from large monogyne colonies were more barrel-shaped (i.e., narrower at the top) in small workers but changed toward heart-shaped more rapidly as workers become larger (i.e., the slope of the regression of HW2/BL on BL for large monogyne colonies (0.242, Figures 2A, B) was significantly greater than those of polygyne and small monogyne colonies (0.173 and 0.158, respectively: Figure 2B, differences in slope shown in Table 1, line 1, column 5; and Table 2, line 1, column 5). This same relationship can also be seen in the slopes in Figure 2A. The result is that the head shapes of workers changed more rapidly to heart-shaped in large monogyne colonies than in small and polygyne colonies.

**Table 1.**
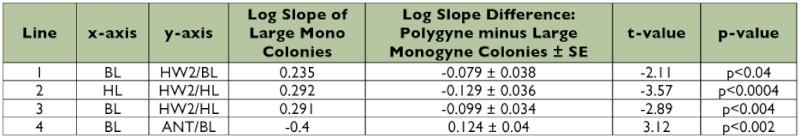
Comparison between slopes of large monogyne and large polygyne colonies. Only variables that were significantly different are shown.

**Table 2.**
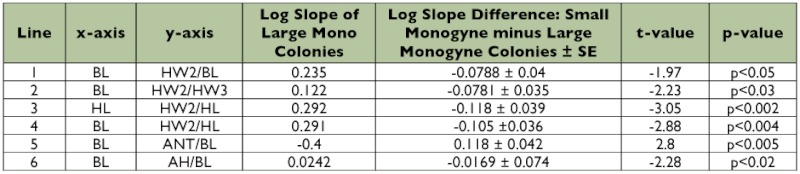
Comparison between slopes of large and small monogyne colonies. Only variables that were significantly different are shown.

**Table 3.**
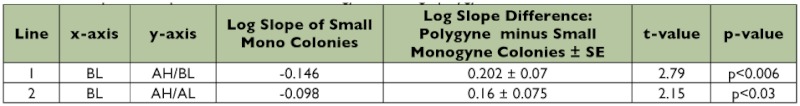
Comparison of parameters for small monogyne and large polygyne colonies.

**Figure 3.**
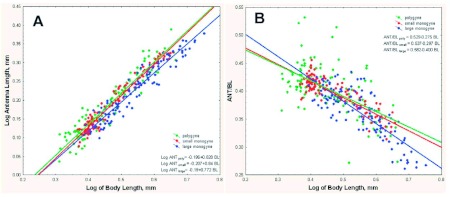
(A) As body size increased, the antenna became relatively smaller, and this negative allometry was greater in monogyne colonies; that is, for a given body size of monogyne worker, the antenna was shorter than for a polygyne one (slopes; 0.82 for polygyne, 0.84 for small monogyne, 0.77 for large monogyne). (B) Plotting the log of ANT-to-BL ratio against BL makes the higher rate of decrease for large monogyne colonies more apparent. High quality figures are available online.

The antennae of workers became relatively shorter as worker size increased, that is, antennae were negatively allometric to body size (Tschinkel et al. 2003) (Appendix 1, line 9; Appendix 2, line 9; Appendix 3, line 11 appendices available online), but colony types differed in the magnitude of this negative allometry. A plot of the logs of the ANT/BL ratio against BL (Figure 3B) shows that the antennae of workers from large monogyne colonies became relatively shorter more rapidly (slope = -0.40) with worker size than did those of polygyne and small monogyne colonies, -0.28 and -0.30, respectively, which did not differ from each other (differences in slopes are shown in column 5 of Table 1, line 4; Table 2, line 5). This same relationship is seen in the lower slope for large monogyne colonies in Figure 3 A. Workers from small monogyne and from polygyne colonies were virtually identical in the relative length of their antennae across the range of worker sizes, whereas workers from large monogyne colonies had shorter antennae on larger workers of similar size.

**Figure 4.**
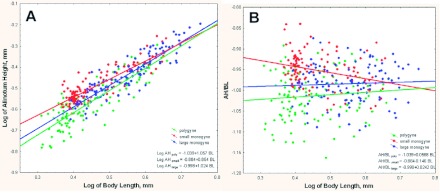
(A) As body size increased, the alinotum heights of workers from large monogyne and polygyne colonies were approximately isometric to body size, but those of small monogyne workers were negatively allometric in an unexpected way—they were more robust than those of the other comparison groups when the workers were small but about the same when the workers were large. The result is a log slope significantly less than 1.0 (0.85), but a higher intercept. B. When the data are plotted as a log ratio of ANT to BL, the isometry of the alinotum height for polygyne and large monogyne workers and the negative allometry of those from small monogyne colonies are clearly shown. High quality figures are available online.

Alinotum height (AH) is an index of the “robustness” of the alinotum shape. Higher values of the AH-to-BL ratio indicate an alinotum that is deeper relative to its own length or the body length (which are isometric to one another). AH was isometric to BL in both large monogyne and polygyne colonies (Figure 4A; slopes = 1.024 and 1.027, respectively; not significantly different from each other or 1.0), and the slopes of the AH/BL ratio on BL were not significantly different from zero (Figure 4B; 0.0242 and 0.057, respectively; Appendix 1, line 15; Appendix 2, line 15). On the other hand, for workers from small monogyne colonies, the slope of the AH/BL ratio on BL was significantly negative (-0.146) (Figure 4B; Appendix 3, line 17), and that of the regression of AH on BL was significantly less than 1.0 (0.854, Figure 4A). Interestingly, the reason was that small workers in small monogyne colonies had a more “robust” alinotum than similar-sized workers in large monogyne and polygyne colonies. The alinotum heights converged at large worker sizes.

**Figure 5.**
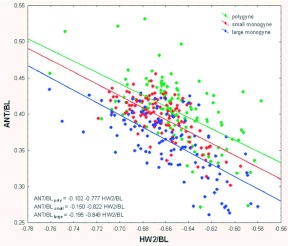
Plotting the ANT-to-BL ratio against the HW2-to-BL ratio shows that, for any given HW2-to-BL ratio, the antennae are relatively the shortest in large monogyne colonies, longer in small monogyne colonies, and longest in polygyne colonies. The slopes of all three regressions are the same (0.78–0.85), and only the intercepts differ (-0.10 polygyne, -1.5 small monogyne, -0.2 large monogyne; —-test; -7.03; p < 0.00001). High quality figures are available online.

Plotting the ANT-to-BL ratio against the HW2-to-BL ratio (Figure 5) showed that for any given HW2-to-BL ratio, the antennae were relatively the shortest in large monogyne colonies, longer in small monogyne colonies, and longest in polygyne colonies. The slopes of all three regressions were the same (about -0.8); only the intercepts differed (-0.10 polygyne; -1.5 small monogyne; -0.2 large monogyne; *t*-test: -7.03; p < 0.00001).

Comparing the intercepts indicated that the ANT-to-BL ratio was about 10–12% greater for workers from small monogyne colonies than for workers of any HW2-to-BL ratio from large monogyne colonies, and the ratios of workers from polygyne colonies were about 10% greater than those from small monogyne colonies. The ratios for all three colony types were in a constant ratio to each other across the entire range of HW2-to-BL ratio, suggesting processes that affect both antennal length and head width in a tightly linked fashion and a fixed difference due to colony type. Most probably, these processes are related to the competitive growth of the respective imaginai discs in the head. The ratios of these head measurements to body length were independent of the ratios of alinotum height to body length, possibly because of the greater distance between them.

## Discussion

In relation to worker size, the proportions of worker heads, antennae, and alinota changed at different rates in large monogyne, small monogyne, and polygyne colonies. The differences were not large, and the question remains whether they are functionally important, but they do suggest that changes in worker shape during growth do not derive only from factors intrinsic to the workers themselves but are influenced by colony level factors. Workers from large monogyne colonies started with lower HW2-to-BL ratios than those from the other colony types but increased more rapidly through a greater range of HW2-to-BL ratios than the others. In other words, their heads started out more barrel-shaped and became heart-shaped more rapidly than those of the other two colony types. Similarly, the antennae of workers from large monogyne colonies become relatively shorter more rapidly than the other colony types as worker size increases. The fact that relative antennal length follows the opposite pattern to HW2 suggests that the imaginai discs for these two dimensions compete for resources, and that investment in the growth of one of them reduces growth in the other.

The allometric differences are probably best described as environmental static allometries, as defined by Shingleton et al. (2007) because they are expressions of similar genotypes in different (social) environments. Of course, polygyne fire ants differ from monogyne at one locus (*Gp-9,* Ross et al. 2003), but this small difference is unlikely to affect polymorphism significantly because, when polygyne colonies are reduced to a single queen, they take on very monogyne characteristics. Although Fjerdingstad and Crozier (2006) suggest that polymorphism is associated with increased genetic diversity (like that, e.g., resulting from polygyny), the opposite is true in *S. invicta*.

The greater similarity of worker allometry between polygyne and small monogyne colonies than with that of large monogyne ones is probably significant. The factor that links the first two and differentiates them from the last is probably colony nutrition, with its echo in individual worker nutrition. Within the life cycle of monogyne colonies, the rate of brood production per worker decreases as colonies grow, so the ratio of workers to brood increases and each larva potentially receives more food and care (Brian, 1953; Markin et al. 1973; Wood and Tschinkel 1981). This greater care and better nutrition in large colonies probably underlies the larger average worker size, higher proportion of major workers, and higher worker fat content in these colonies (Tschinkel 1993, 2006). In contrast, although polymorphism is present in polygyne colonies (Figure 1; Greenberg et al, 1985), the large numbers of eggs laid by the multiple queens cause the worker-to-brood ratios to remain low, resulting in workers that are smaller, on average, than those from monogyne colonies of similar size. Small monogyne colonies also have high worker-to-brood ratios, so they are more similar to polygyne colonies than to large monogyne ones.

Our data accord with the predictions of the imaginai disk—interaction growth model proposed by Nijhout and Wheeler (1996), in which a fixed supply of resources during metamorphosis results in competition among imaginai disks, resulting in asymmetrical development of body parts in holometabolous insects. The outcome of this competition may therefore differ with the total amount of resources available within the worker, and these may in turn depend on the social form and colony size, as in the paragraph above. Small monogyne and large polygyne colonies are probably more similar to each other in nutritional status than to large monogyne ones because their worker-to-brood ratios are more similar. This similar nutritional status may in turn have similar effects on the development of their imaginai discs. The size of an insect's component organs (as well as its total size) depends on the hormonally determined critical size of each imaginai disc as well as how long and how fast each grows after reaching critical size and before reaching the termination of growth (Shingleton et al. 2007). As with body size, nutrition is thought to affect the final size of organs by influencing the imaginai disc size at critical size and the duration and rate of a disc's final growth after it reaches critical size. The developing imaginai discs may also have different reaction norms to nutritional status and/or interact with each other to influence each other's final sizes (Shingleton et al. 2007).

In light of the imaginal-disk-competition hypothesis, the proximity of imaginai discs may explain why the allometries within the head (antennae and head width 2) remain in constant relationship to each other within each colony type (Figure 5) while that of alinotum height, the alinotum being distant from the head imaginai discs, seems independent of these head allometries (Figure 4).

Alinotum shape is isometric to worker size in polygyne and large monogyne colonies but not in small monogyne colonies. In these last, the greater robustness of the alinotum in small workers and its lesser robustness in large workers is odd, as it is opposite to the usual trend. Perhaps this is, after all, an interaction with the imaginai discs in the head. Whether it has functional importance is unknown.

## Abbreviations

AL: alinotum length;
AH: alinotum height;
HL: head length;
HWI: head width across the eyes;
HW2: head width above the eyes, ca. 75% of the distance from the clypeus to the apex of the head;
HW3: head width below the eyes, ca. 25% of the distance from the clypeus to the head apex;
FL: flagellum length;
SC: scape length;
AN: antennal length (FL + SC);
GL: length of first gaster segment;
GW: width of first gaster segment;
FE(1,2,3): femur length;
OLS: ordinary least squares regression;
RMA: reduced major axis regression;
TB(1,2,3): tibia length;
TA(1,2,3): tarsus length;
LG(1,2,3): total leg length (FE +TB + TA);
PL(1,2): petiole length;
PH(1,2): petiole height;
WT: weight (mg);
BL: total body length (HL + AL +PL1 + PL2 + GL)
